# Gene differential co-expression analysis of male infertility patients based on statistical and machine learning methods

**DOI:** 10.3389/fmicb.2023.1092143

**Published:** 2023-01-27

**Authors:** Xuan Jia, ZhiXiang Yin, Yu Peng

**Affiliations:** School of Mathematics, Physics and Statistics, Shanghai University of Engineering Science, Shanghai, China

**Keywords:** male infertility, hypergeometric distribution, Fisher test, Gibbs sampling, machine learning, gene interaction network, HPV

## Abstract

Male infertility has always been one of the important factors affecting the infertility of couples of gestational age. The reasons that affect male infertility includes living habits, hereditary factors, etc. Identifying the genetic causes of male infertility can help us understand the biology of male infertility, as well as the diagnosis of genetic testing and the determination of clinical treatment options. While current research has made significant progress in the genes that cause sperm defects in men, genetic studies of sperm content defects are still lacking. This article is based on a dataset of gene expression data on the X chromosome in patients with azoospermia, mild and severe oligospermia. Due to the difference in the degree of disease between patients and the possible difference in genetic causes, common classical clustering methods such as k-means, hierarchical clustering, etc. cannot effectively identify samples (realize simultaneous clustering of samples and features). In this paper, we use machine learning and various statistical methods such as hypergeometric distribution, Gibbs sampling, Fisher test, etc. and genes the interaction network for cluster analysis of gene expression data of male infertility patients has certain advantages compared with existing methods. The cluster results were identified by differential co-expression analysis of gene expression data in male infertility patients, and the model recognition clusters were analyzed by multiple gene enrichment methods, showing different degrees of enrichment in various enzyme activities, cancer, virus-related, ATP and ADP production, and other pathways. At the same time, as this paper is an unsupervised analysis of genetic factors of male infertility patients, we constructed a simulated data set, in which the clustering results have been determined, which can be used to measure the effect of discriminant model recognition. Through comparison, it finds that the proposed model has a better identification effect.

## 1. Introduction

For a long time, infertility has been a difficult problem for many couples of gestational age. With the increase of life pressure, infertility is increasing every year. About 15% of gestational age couples suffer from infertility symptoms of varying degrees, of which about 50% are caused by male infertility (Dada et al., 2003). About 7% of men in the general population suffer from different degrees of infertility. The causes of male infertility are related to many influencing factors, including different diseases, genetics, living habits and other factors that may cause or interact to cause male infertility. Although men with this disorder cannot pass on their genetic information naturally, genetic factors can still contribute to male infertility. In approximately 15% of infertile men a genetic defect is most likely the underlying cause of the pathology (Tournaye et al., 2017; Krausz and Riera-Escamilla, 2018). For example, autosomal recessive or X-linked male infertility mutations transmitted by normal parents can cause infertility (Chillón et al., 1995; Yatsenko et al., 2015). Genetic causes have also been found to have an important role in severe male infertility, such as severe oligospermia (<5 million sperm cells per milliliter) or azoospermia (azoospermia in ejaculation; Lopes et al., 2013; Krausz and Riera-Escamilla, 2018). Identifying the genes responsible for male infertility is important for increasing our understanding of the biology of the disease and for genetic testing for diagnosis and clinical treatment. Genes such as NLRP3, BRD7 and others have been shown to affect male fertility (Aquila et al., 2004; Wang et al., 2016; Antonuccio et al., 2021). At the same time, with the rapid development of genetics, more than 3,000 genetic diseases have been discovered, of which about 250 are only found in men, and women have no or little disease. Because women have two X chromosomes, the pathogenic gene on one X chromosome can often be masked by the normal gene on the other X chromosome, so they do not show symptoms. Men, on the other hand, have only one X chromosome. If there is a disease-causing gene on it, there is no corresponding normal gene to cover up, resulting in the disease. In recent years, with the deepening of research, there are about 521 genes that cause male infertility in different forms (Xavier et al., 2021), many of which are related to the X chromosome, such as mouse androgen receptor gene mutation, through chain reaction mapping The X chromosome leads to infertility in mice (Lyon et al., 1970), and there is one more X chromosome in males, that is, the sex chromosome is XXY (Jacobs and Strong, 1959) and so on.

Many scholars have carried out various experimental methods to study the genetic causes of male infertility. Through RNA interference or knockout experiments, the gene cannot be expressed normally, and whether the target abnormality occurs in cells or individuals is observed, and whether the gene is related to the cause of the disease is detected. However, experimental methods are generally time-consuming, labor-intensive, and expensive, and experimental methods are generally designed in a targeted manner on the premise that the experimenter obtains genes that may have basic interference. Technological advances and methodological developments in genomics are critical for identifying genetic factors in male infertility.

In this paper, we use a data set covering all gene expression levels of the male X chromosome in the GEO database, the Gene Expression Omnibus (GEO), a public database that contains 659,203 gene sample data from 9,528 different platforms (Ron et al., 2002). And based on a variety of statistical methods and machine learning analysis of gene expression data of male infertility patients, to identify groups of interacting gene clusters that may contribute to male infertility of various phenotypes in various ways. Common hierarchical clustering, k-means and other clustering algorithms are clustering under the assumption that all samples have certain characteristics, and the cluster data of the identified clusters have the same characteristics in all samples. However, the expression of gene data is affected by different sampling individuals, different tissues of the same individual, etc., resulting in different expression of measured gene data in different samples, and common clustering algorithms cannot meet the identification of differential gene expression modules (implementation basis Partial samples of gene expression data to partition gene sample data). For the identification of differentially co-expressed modules, a biclustering algorithm can be used to screen functionally related genes, genes involved in the same pathway, and genes affected by the same drug or a pathological condition. The biclustering algorithm was first proposed in Hartigan (1972), is a two-dimensional data mining technique that allows simultaneous clustering of rows (representing genes) and columns (representing samples/conditions) in a gene expression matrix. Developments continued in the following decades, with (Cheng and Church, 2000; Lazzeroni and Owen, 2000; Bergmann et al., 2003; Kluger et al., 2003; Chiu et al., 2004; Prelić et al., 2006; Dhollander et al., 2007; Gu and Liu, 2008; Li et al., 2009; Hochreiter et al., 2010; Madeira et al., 2010; Medina et al., 2010; Chen et al., 2011; De Smet and Marchal, 2011; Zhao et al., 2011; Zhou et al., 2012; Goncalves and Madeira, 2014; Henriques and Madeira, 2016a,b; Alzahrani et al., 2017; Guo et al., 2021) being articles on different clustering algorithms. Among them, BCPlaid (Lazzeroni and Owen, 2000), QUBIC (Li et al., 2009), C&C (Cheng and Church, 2000), FABIA (Hochreiter et al., 2010) are the more popular biclustering algorithms. Genomics data analysis clustering using machine learning, deep learning, etc., for identifying cell subpopulations, genomic analysis, etc.(Jiang et al., 2020; Lazareva et al., 2020; Peng et al., 2020; Gerniers et al., 2021; Peng et al., 2021; Yi et al., 2021; Peng et al., 2022; Zhai et al., 2022). Analysis of bronchoalveolar immune cells in COVID-19 patients based on genetic data (Liao et al., 2020). By processing the GSE37948 data set (Krausz et al., 2012), which contains expression levels of gene data on the X chromosome in testicular tissue from patients with varying degrees of infertility, we identified 19 distinct double clusters, indicating the existence of multiple double clusters identified in this paper there are multiple enriched pathways and there are functional and organizational correlations between the enriched pathways. And the performance of the method is verified using a data set similar to the real gene expression level.

## 2. Materials and methods

### 2.1. Methods

Rank-rank hyper geometric overlap (RRHO; Plaisier et al., 2010) uses unsupervised learning to sort the gene expression profile data of two samples of different categories, and uses hyper geometric distribution to iteratively calculate the *p*-values of all combinations to find the optimal overlap gene combination. In this paper, the sample expression data of two different genes is brought into the RRHO method to find the optimal overlapping sample set, and the SNR value of the signal-to-noise ratio of the sample gene set is calculated to determine whether the clusters have differential expression. For a single gene in the sample set, the SNR value is defined as:


$$SNR\left(\mathrm{g,}P^{\prime }\right)=\frac{\mu_{g,P^{\prime }}-\mu_{g,\bar{P^{\prime }}}}{\sigma_{g,P^{\prime }}+\sigma_{g,\bar{P^{\prime }}}}$$

$\mu_{g,P^{\prime }}$ , $\mu_{g,\bar{P^{\prime }}}$ are the mean in the delimited sample set $P^{\prime }$ and the mean in the data outside the sample set, respectively. $\sigma_{g,P^{\prime }}$ , $\sigma_{g,P^{\prime }}$ represent the standard deviation of the data in the corresponding set. The overall signal-to-noise ratio of the cluster is the average of the signal-to-noise ratios of individual genes in the sample set.

**Figure 1:**
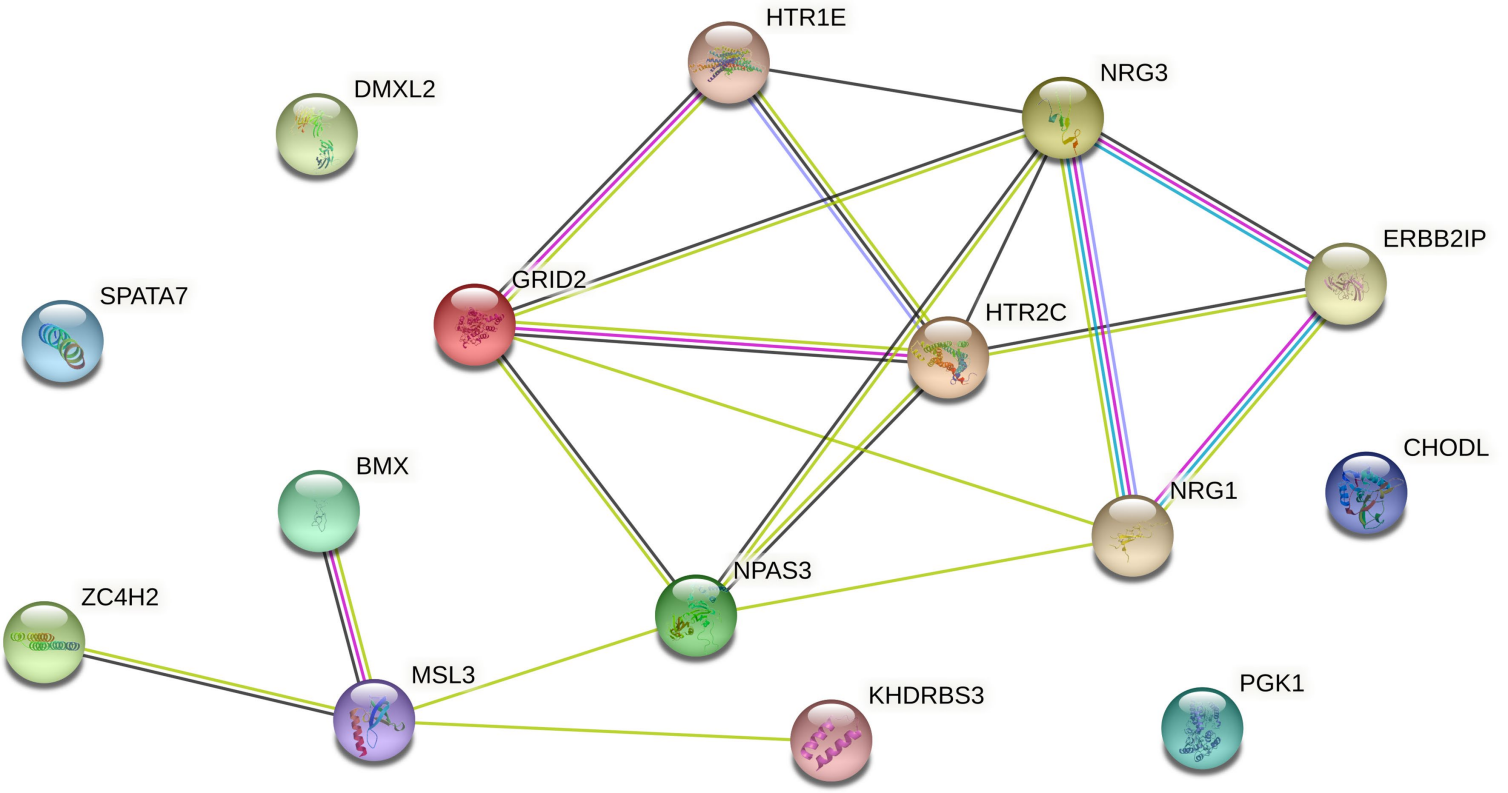
Interaction network of some genes in GDS37948.

**Figure 2:**
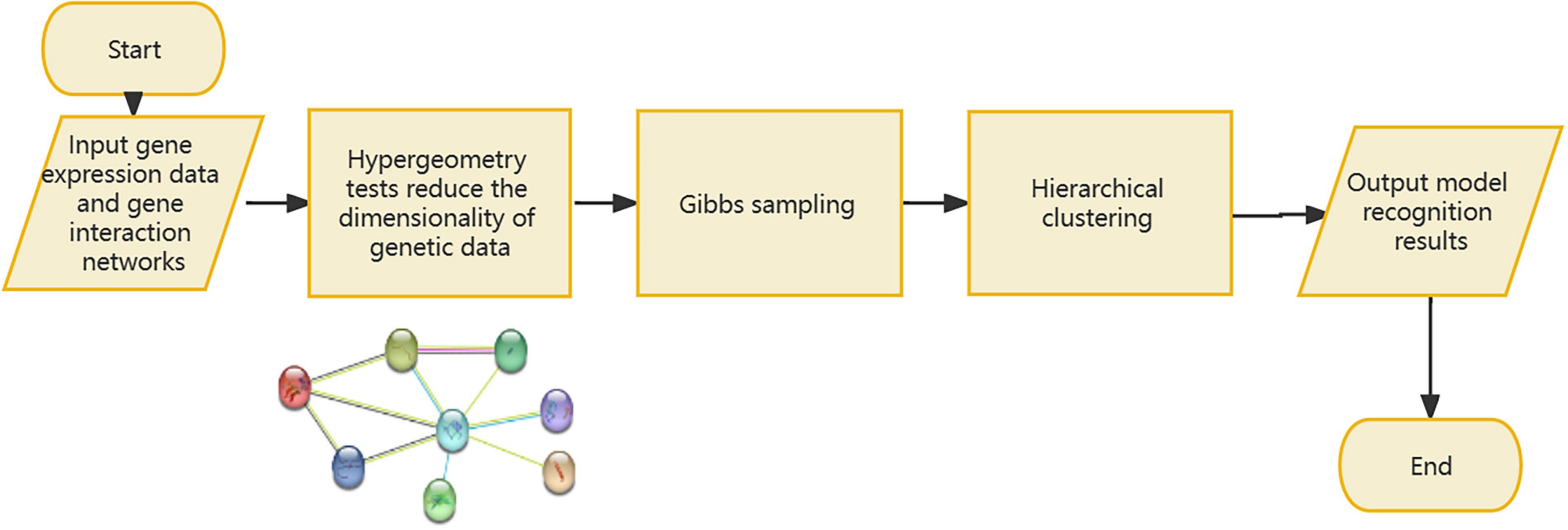
Introduction to the model process.

If the signal-to-noise ratio value of the identified sample and gene set is greater than the specified threshold, the set will be retained, and the corresponding genome is considered to have a relationship with the gene data. If one gene cannot form a relationship with other genes in the data, it will be discarded in the subsequent processing, so as to realize the dimensionality reduction processing of the gene data. However, since the genes known to be associated with disease from Ghiassian et al. (2015) form a compact but not tightly connected subgraph on the PPI, this paper does not loop through all the genes in the data set, but adds a gene interaction network to the data processing. Using the String database, there is known and predicted gene-protein interaction networks in the database. In this paper, the genes involved in the data set are searched for the interaction network, and the isolated gene points are discarded. The genes existing in the gene network are combined in pairs, and the hierarchical clustering method is used for preliminary clustering to assist in determining the default set signal-to-noise ratio threshold. The set of gene samples constructed by preliminary clustering is calculated as the average of the signal-to-noise ratio values in all sets, and 1/2 of this mean is used as the threshold. When the signal-to-noise ratio of the gene sample set constructed by the RRHO method is used. If the ratio is greater than this threshold, the gene is retained and a new set of double clusters is obtained. Otherwise, in the gene network, the connected edges are discarded. Due to the large number of genes, a partial gene network is shown in Figure 1. Figure 2 briefly depicts the model’s approach. The interrelation data of all genes are presented in Supplementary Table 1.

Since only gene pairs and their corresponding sample sets can be obtained after using the RRHO method, Gibbs sampling (Sheng et al., 2003) is used for the data processed in the first step to make assumptions about the distribution of gene sample data to merge gene clusters. The statistical assumptions for sampling are as following:


$$x_{\mathrm{ji}}|\theta_{\mathrm{ic}},s_{j}∼\mathrm{Bernoulli}\left(x_{\mathrm{ji}}|\theta_{{\mathrm{is}}_{j}}\right)$$

$$\begin{array}{l} s_{j}|m∼\mathrm{Categorical}\left(s_{j}|m\right)\\ \;\;\;\;\;\;\;\theta_{\mathrm{ic}}∼\mathrm{Beta}\left(\alpha /2\right)\\ \;\;\;\;m∼\mathrm{Dirichlet}\left(\beta /K\right). \end{array}$$

i represents the gene, j represents the sample, if the association exists after step 1, $x_{\mathrm{ji}}$ is assigned 1 else it is 0. $s_{j}$ represents which module the gene edge j belongs to, through the calculation of the edge transition probability in Gibbs sampling:


$$\begin{array}{l} P(s_{j}=\;\;\;k|\mathrm{X,}\;s_{-j};\alpha ,\beta )\propto \\ \;\;\;\;\;\;\;\;\;\;\;\;\;\;\;\;\;\times \prod_{i:x_{\mathrm{ji}}=1}\left[\frac{\alpha /2+\sum_{l:s_{l}=k,l\neq j}x_{\mathrm{li}}}{\alpha +\left|\left\{l:s_{l}=\mathrm{k,l}\neq j\right\}\right|}\right]\\ \;\;\;\;\;\;\;\;\;\;\;\;\;\;\;\;\;\times \frac{\left|\left\{l:s_{l}=\mathrm{k,l}\neq j\right\}\right|+\beta /K}{n-1+\beta }\left[\frac{\alpha /2+\sum_{l:s_{l}=k,l\neq j}\left(1-x_{\mathrm{li}}\right)}{\alpha +\left|\left\{l:s_{l}=\mathrm{k,l}\neq j\right\}\right|}\right] \end{array}$$

Among them, k is set to the number of clusters retained after the calculation and processing of the RRHO method. Finally, the statistical part of Gibbs sampling assumes that the data has a certain prior distribution involving parameter α and β, but because the genetic data lacks the corresponding statistical research foundation, the parameter α and β are set as hyperparameters. At the end of data processing, Fisher’s exact test is used to process the calculated set data again, and the sample data in the two clusters are processed to calculate its value of *p*. The set threshold is used to determine whether there is a significant difference between the two sets, and the genes in the two sample sets without significant differences are merged, and the sample data of the corresponding gene is taken out and brought into the hierarchical clustering, and the number of clusters is 2. Since a gene is up-regulated in half of the samples, it will be differentially expressed in the remaining part, so, we limit samples in clusters to less than 55% of the total number of samples in the data set as a difference in the gene set. At the same time, in order to limit that the cluster is differentially expressed in the whole data, the SNR value of the newly formed cluster is required to be greater than the threshold value. Otherwise it will not be merged. All the identified clusters are merged cyclically until no new clusters are generated.

### 2.2. Datasets

#### 2.2.1. Male infertility gene expression data

First, the corresponding gene expression data were obtained from the micro array gene expression database. In this paper, the GSE37948 (Krausz et al., 2012) gene expression data set was selected. This data set contains relevant gene expression data of 96 patients with different degrees of infertility, including 74 cases of azoospermia, 6 cases of mild oligozoospermia, and 16 cases of severe oligozoospermia. Excluding known causes of impairing spermatogenesis in patients, gene expression data identification was performed using testicular tissue from 47 men, and KNN nearest neighbor algorithm was used to impute missing values in gene expression profile data while normalizing data for each gene, to remove the effect of different units on the data. The GSE37948 data set contains 1855 genes and gene-identified expression data from 200 male sperm samples. The genes identified therein to cover the entire X chromosome. The related gene network based on the GSE37948 data set was extracted from the String database. Specific gene interaction data are shown in the Supplementary Table: Interrelation data among genes.

#### 2.2.2. Synthetic datasets

Since the method in this paper belongs to unsupervised learning, there are no standard results for the study of male infertility-related genes, so we constructed simulation data similar in structure to GSE39748. The GSE37948 data set has a total of 1,855 genes and 200 samples, but the size of the double-cluster deletion is unknown. To this end, simulated data of 20 known differentially expressed modules were constructed with gene and sample dimensions of 2,000 and 200, respectively. Based on previous research (Prelić et al., 2006; Eren et al., 2013), we can generate simulation data according to the following rules: Genes and sample numbers are sampled from (100, 50, 20, 10, 5) and (100, 50, 20, 10) respectively, the data within the cluster is sampled from N (2, 1), and the rest of the data are sampled from N (0, 1) and allow the intersection of different clusters. Simulated data is used to determine hyperparameters and statistics are used to evaluate clustering results. Since the gene interaction network graph used in the gene data processing corresponds to the gene interaction graph with certain connectivity, we correspondingly construct the connected network graph according to the determined clustering data. Studies have shown that in the gene interaction network, genes related to disease can form compact linker maps (Ghiassian et al., 2015), so we use the method proposed in Bollobás et al. (2003) to construct the network diagram, which can construct a reasonable gene network connection map according to the clustering modules in the expression data.

## 3. Results

### 3.1. Experimental results of male infertility-related gene expression data

By processing the GSE37948 data set, which contains expression levels of gene data on the X chromosome in testicular tissue from patients with azoospermia, mild and severe oligozoospermia. We identified 19 distinct double clusters. There are multiple enriched pathways and there are functional and organizational correlations between the enriched pathways. The hypergeometric test involved in the RRHO method, in which the significance index is adjusted from the set (0.01, 0.05), and the parameter α and β/k involved in the statistical hypothesis in Gibbs sampling are adjusted from the set (5.0, 1.0, 0.5, 0.1) and (100, 1.0, 0.01), respectively. According to the recognition effect of the model on the simulated data set, the final parameters *p* = 0.01, *α* = 0.5, and β/k = 1.0 were determined. The data processed based on the GSE39748 data is brought into the model to identify the gene sample module, and the results were analyzed using a variety of biometric indicators Includes: Disease (OMIN_DISEASE, UP_KW_DISEASE), Functional_Annotations (COG_ONTOLO, UP_KW_BIOLOGICAL_PROCESS, UP_KW_CELLOULAR_COMPONENT, UP_KW_MOLECULAR_FUNCTION, UP_KW_PTM, UP_SEQ_FEATURE), Protein_Domains (INTERPRO, PIR_SUPERFAMILY, SMART, UP_KW_DOMAIN), Gene_Ontology (GOTERBP, CC, MF), Interactins (UP_KW_LIGAND), Pathways (KEGG_PATHWAY, BBID,BIOCARTA), Protein_Domains (INTERPRO, PIR_SUPERFAMILY, SMART, UP_KW_DOMAIN).

Corresponding to the Enrichment analysis results with the cluster id of 1 in Table 1, there were four significantly enriched pathways after analysis by GO and KEGG, two of which were associated with proteins of the autism spectrum, which includes different phenotypic manifestations such as classic autism, Asperger’s syndrome, childhood disintegration Sexual disorder, Rett’s syndrome, and pervasive developmental disorder not otherwise specified. Also significantly enriched into axons, the site of neurotransmitter storage and release. And outside the cytoplasmic membrane, referring to gene products attached to the plasma membrane or cell wall.

**Table 1:** Clustering results identified in the statistical method proposed in this paper based on the GDS37948 male infertility data set.

ID	avgSNR	Number of samples	Number of samples
1	0.700870148	13	56
2	0.816555484	3	110
3	0.775713429	3	88
4	0.745638081	8	101
5	0.743384851	3	72
6	0.743381552	4	71
7	0.730139247	351	20
8	0.718222619	6	110
9	0.716803164	3	91
10	0.70627255	3	101
11	0.703721749	3	68
12	1.15234204	482	12
13	0.678448517	6	95
14	0.678084094	11	103
15	0.67773126	25	110
16	0.674885829	3	38
17	0.671869245	6	92
18	0.668664873	3	84
19	0.667155842	3	49

Corresponding to the Enrichment analysis results with the cluster id of 2 in Table 1, enriched in chemical synaptic transmission, cell membrane, and plasma membrane pathways. Release of neurotransmitter molecules from presynaptic vesicles across chemical synapses followed by post synaptic activation of neurotransmitter receptors on target cells (neurons, muscles, or secretory cells), and the effect of this activation on synapses Post-membrane potential and ionic composition of the post synaptic cytoplasm. This process includes spontaneous and evoked release of neurotransmitters and all parts of synaptic vesicle exocytosis. Evoked transmission begins when the action potential reaches the presynaptic.

Corresponding to the Enrichment analysis results with the cluster id of 3 in Table 1, by SMART, INTERPRO, UP_KW_DOMAIN showed enrichment to the SH3 domain. The SH3 (src homology-3) domain is a small protein module containing approximately 50 amino acid residues. They are present in a variety of intracellular or membrane-associated proteins, for example, in a variety of proteins with enzymatic activity, in adaptor proteins such as fodrin and the yeast actin-binding protein ABP-1. The SH3 domain has a characteristic fold, which consists of five or six β-strands arranged in two tightly packed antiparallel β-sheets. The linker region may contain short helices. The surface of the SH3 domain bears a flat hydrophobic ligand-binding pocket consisting of three shallow grooves defined by conserved aromatic residues in which the ligands are arranged in an extended left-handed helix. Ligands bind with low affinity, but this can be enhanced by multiple interactions. The region bound by the SH3 domain is proline-rich in all cases and contains PXXP as a core conserved binding motif. The function of SH3 domains is unclear, but they may mediate many different processes, such as increasing the local concentration of proteins, changing their subcellular location and mediating the assembly of large multiprotein complexes.

Through enrichment analysis, we found that the gene sets of the identified clusters were enriched in a variety of enzyme activities, ADP and ATP related generation reactions, replication and translation of genetic material DNA and RNA, neurotransmitter transmission links and other pathways. Multiple clusters were enriched in RNA polymerase II forward and transcriptional regulatory pathways, protein tyrosine related enzyme pathways, neural synapses, neurotransmitter transmission links, ATP, ADP synthesis related links. There were two clusters of gene sets enriched to human papillomavirus infection pathway. One cluster was significantly enriched in calcium ion related pathways. Another cluster was significantly enriched in the inositol phosphate metabolism pathway. SH3 (src Homology-3) domains, proteoglycan cancer pathway, PDZ domain, Hippo signaling pathway, Tight junction pathway, PB1 domain and other pathways were also enriched in some clusters. Each cluster enriched in the above described pathways at the same time there are other enrichment pathways with different functions. There may be multiple gene interactions enriched in different pathways leading to differences in sperm motility.

**Figure 3:**
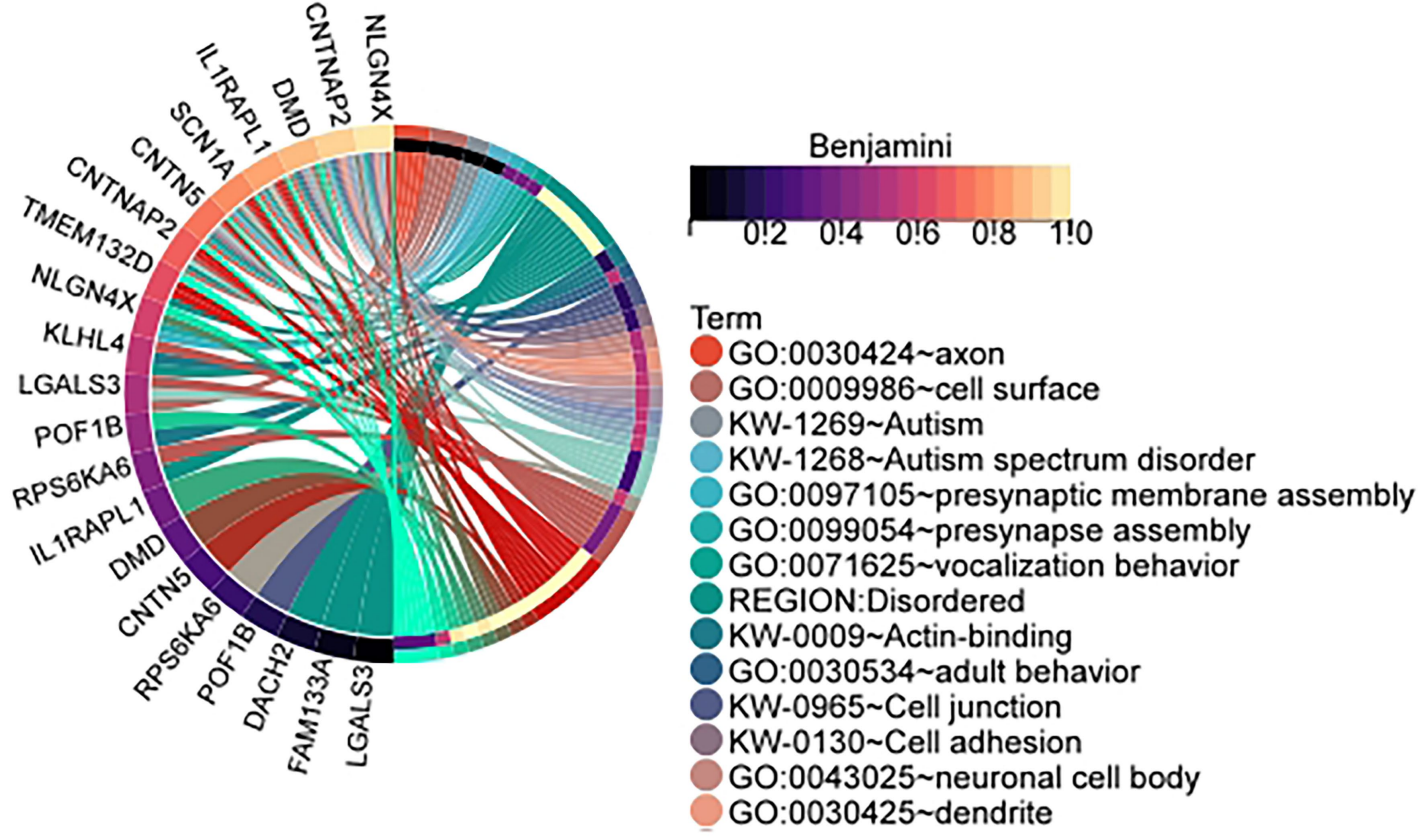
Enrichment circle plot of genes in clusters identified by our method in the male infertility data set. The cluster is the id in Table 1: 1. (Visualization of the relationship between genes and enrichment pathways).

In order to determine whether the data is significantly enriched, the *p*-values of the enrichment results are corrected using the Benjamini method and the Bonferroni method. The specific identified differentially expressed genes and the number of samples is shown in Table 1. Specific gene and sample data are included in the Supplementary Table: The result of identification. Table 2 is the cluster-related enrichment results, Figure 3 visualizes the correlation enrichment results, and the enrichment analysis results of all clusters are shown in Supplementary Data.

**Table 2:** Enrichment results of genes in a cluster identified by our method in the male infertility data set.

Category	Term	Genes	Bonferroni	Benjamini
GOTERM_CC_DIRECT	GO:0030424 ~ axon	CNTNAP2, CNTN5, IL1RAPL1, DMD, SCN1A	0.002330526	0.002333212
GOTERM_CC_DIRECT	GO:0009986 ~ cell surface	LGALS3, CNTNAP2, NLGN4X, IL1RAPL1, DMD	0.021009445	0.010615268
UP_KW_DISEASE	KW-1269 ~ Autism	CNTNAP2, NLGN4X, SCN1A	0.002854718	0.002858289
UP_KW_DISEASE	KW-1268 ~ Autism spectrum disorder	CNTNAP2, NLGN4X, SCN1A	0.014578999	0.007336422

Only the pathways and related parameters that were modified and significantly enriched by Bonferroni and Benjamini are listed in the table. The cluster is the id in Table 1: 1.

### 3.2. Simulation data experimental results

Since this paper belongs to unsupervised learning, there is no standard answer for the quantitative study of male sperm motility. At the same time, in order to better determine the value of hyper-parameters in the statistical method used in this paper, simulated data similar to gene expression profile datasets are constructed to be used in the method proposed in this paper. The clustering results in the simulated data have been determined and can be used to evaluate the model performance. Comparing the identification results of the simulated data set with the results of similar methods, and the results show that the model proposed in this paper may have higher accuracy in the analysis of genetic factors in the quantitative study of male sperm (Table 3).

**Table 3:** The jaccard similarity coefficient between the clustering results identified by the three methods on different simulated datasets and the real clusters, where simulation data represents (the number of samples, the number of genes).

Simulation data	BCPlaid	C&C	COEXSML (this work)
(10, 5)	0.0000	0.0000	0.0346
(10, 10)	0.0000	0.0002	0.1089
(10, 20)	0.0000	0.0005	0.0552
(10, 50)	0.0003	0.0012	0.1150
(10, 100)	0.0002	0.0022	0.2023
(20, 5)	0.0000	0.0001	0.4509
(20, 10)	0.0000	0.0005	0.6126
(20, 20)	0.0000	0.0009	0.5373
(20, 50)	0.0004	0.0023	0.3382
(20, 100)	0.0012	0.0033	0.3195
(50, 5)	0.0000	0.0003	0.5112
(50, 10)	0.0000	0.0013	0.7917
(50, 20)	0.0020	0.0033	0.8291
(50, 50)	0.0000	0.0047	0.8715
(50, 100)	0.0024	0.0061	0.8097
(100,5)	0.0000	0.0004	0.5123
(100, 10)	0.0000	0.0042	0.6277
(100, 20)	0.0000	0.0038	0.6794
(100, 50)	0.0000	0.0074	0.6938
(100, 100)	0.0007	0.0214	0.5455

**Figure 4:**
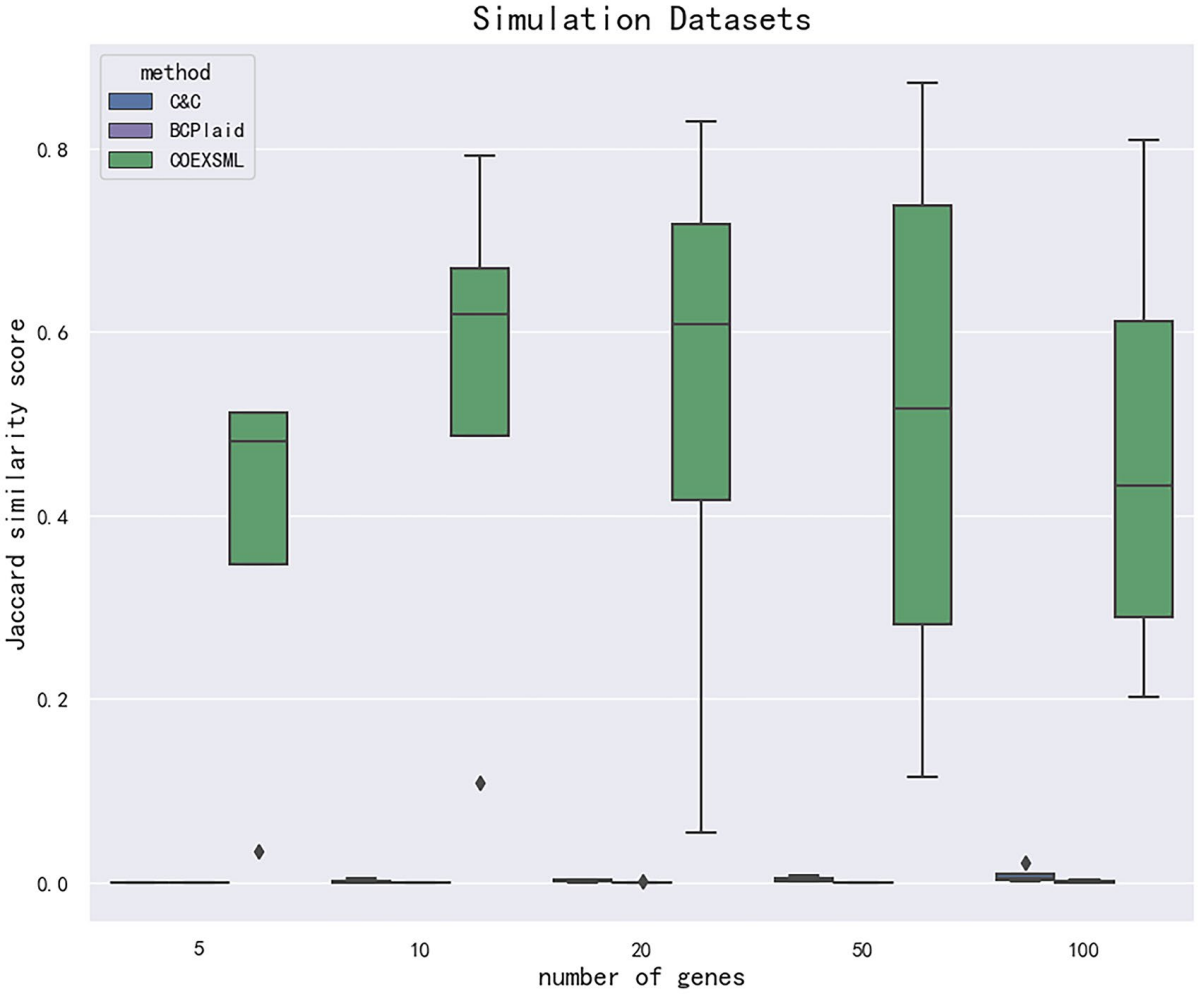
The data are divided according to the difference in the number of genes in the clusters in the simulated data set. The clustering effect is measured according to the jaccard similarity coefficient, and compared with other methods. COEXSML is the method proposed in this paper.

To identify the differential expression module of the simulated data, we used the C&C (Cheng and Church, 2000) and BCPlaid (Lazzeroni and Owen, 2000) methods to cluster the data, and calculated the jaccard similarity coefficient of the results, which was often used to compare the similarity and difference between the limited sample sets, among which the jaccard coefficient. The higher the value, the higher the similarity between sets. The stable parameters were tuned best in each model. The specific results are shown in Supplementary Table 3, and the corresponding box plot is in Figure 4.

## 4. Conclusion

Based on the analysis of the GSE37948 male infertility-related gene detection data set in the GEO database, this paper proposes a bicluster analysis method based on hypergeometric distribution, Gibbs sampling and machine learning, and establishes simulation data similar to the GSE37948 data set. The common bicluster analysis methods C&C (Cheng and Church, 2000) and BCPlaid (Lazzeroni and Owen, 2000) have compared the experimental results. The results show that the method proposed in this paper has a higher accuracy in the identification of biclusters on the established simulation data set.

Through enrichment analysis, we found that the gene sets of the identified clusters were enriched in a variety of enzyme activities, ADP and ATP related generation reactions, replication and translation of genetic material DNA and RNA, neurotransmitter transmission links and other pathways. Multiple clusters were enriched in RNA polymerase II forward and transcriptional regulatory pathways, protein tyrosine related enzyme pathways, neural synapses, neurotransmitter transmission links, ATP, ADP synthesis related links. There were two clusters of gene sets enriched to human papillomavirus infection pathway. One cluster was significantly enriched in the inositol phosphate metabolism pathway. Each cluster enriched in the above described pathways at the same time there are other enrichment pathways with different functions. There may be multiple gene interactions enriched in different pathways leading to differences in sperm motility.

Infertility is a complex pathological condition that presents with a wide range of heterogeneous prototypes, and identifying the genes that cause male infertility is important to increase our biological understanding and clinically relevant treatments. The genetic causes of male infertility are chromosomal abnormalities, gene mutations and other reasons, which may be present in autosomes or in sex chromosomes, considering the particularity of male infertility, this article only considers the study of related genes on the X chromosome. With the development of genetic testing technology, the relevant data has increased significantly, and follow-up research can fully explore the information contained in the gene expression data of relevant patients from more aspects.

## Data availability statement

The original contributions presented in the study are included in the article/Supplementary material, further inquiries can be directed to the corresponding author.

## Author contributions

XJ proposed the model and completed the manuscript writing. YP assisted in completing the model construction. YP and ZY reviewed and revised the manuscript. ZY provided financial support. All authors contributed to the article and approved the submitted version.

## Funding

This research was supported by the National Natural Science Foundation of China (No: 62072296).

## Conflict of interest

The authors declare that the research was conducted in the absence of any commercial or financial relationships that could be construed as a potential conflict of interest.

## Publisher’s note

All claims expressed in this article are solely those of the authors and do not necessarily represent those of their affiliated organizations, or those of the publisher, the editors and the reviewers. Any product that may be evaluated in this article, or claim that may be made by its manufacturer, is not guaranteed or endorsed by the publisher.
