# Supplementary material for: Gene differential co-expression analysis of male infertility patients based on statistical and machine learning methods
Source: Front Microbiol. 2023 Jan 27;14:1092143. doi: 10.3389/fmicb.2023.1092143 (PMC9911419; doi:10.3389/fmicb.2023.1092143)
Supplement: Supplementary file 2 [file Data_Sheet_1.docx]

Supplementary Material

# Supplementary Data

The order corresponds to the ids in Supplementary Table :Enrichment results of multiple pathways

**00**

GO:0004714

transmembrane receptor protein tyrosine kinase activity

Molecular Function

Definition

Combining with a signal and transmitting the signal from one side of the membrane to the other to initiate a change in cell activity by catalysis of the reaction: ATP + a protein-L-tyrosine = ADP + a protein-L-tyrosine phosphate.

GO:0007268

chemical synaptic transmission

Biological Process

Definition

The vesicular release of classical neurotransmitter molecules from a presynapse, across a chemical synapse, the subsequent activation of neurotransmitter receptors at the postsynapse of a target cell (neuron, muscle, or secretory cell) and the effects of this activation on the postsynaptic membrane potential and ionic composition of the postsynaptic cytosol. This process encompasses both spontaneous and evoked release of neurotransmitter and all parts of synaptic vesicle exocytosis. Evoked transmission starts with the arrival of an action potential at the presynapse.

GO:0000118

histone deacetylase complex

Cellular Component

Definition

A protein complex that possesses histone deacetylase activity.

GO:0035556

intracellular signal transduction

Biological Process

Definition

The process in which a signal is passed on to downstream components within the cell, which become activated themselves to further propagate the signal and finally trigger a change in the function or state of the cell.

GO:0003677

DNA binding

Molecular Function

Definition

Any molecular function by which a gene product interacts selectively and non-covalently with DNA (deoxyribonucleic acid).

GO:0098978

glutamatergic synapse

Cellular Component

Definition

A synapse that uses glutamate as a neurotransmitter.

GO:0045944

positive regulation of transcription by RNA polymerase II

Biological Process

Definition

Any process that activates or increases the frequency, rate or extent of transcription from an RNA polymerase II promoter.

GO:0006338

chromatin remodeling

Biological Process

Definition

A dynamic process of chromatin reorganization resulting in changes to chromatin structure. These changes allow DNA metabolic processes such as transcriptional regulation, DNA recombination, DNA repair, and DNA replication. PMID:12042764 PMID:12697820

GO:0050804

modulation of chemical synaptic transmission

Biological Process

Definition

Any process that modulates the frequency or amplitude of synaptic transmission, the process of communication from a neuron to a target (neuron, muscle, or secretory cell) across a synapse. Amplitude, in this case, refers to the change in postsynaptic membrane potential due to a single instance of synaptic transmission.

GO:0007399

nervous system development

Biological Process

Definition

The process whose specific outcome is the progression of nervous tissue over time, from its formation to its mature state.

GO:0045893

positive regulation of DNA-templated transcription

Biological Process

Definition

Any process that activates or increases the frequency, rate or extent of cellular DNA-templated transcription.

GO:0005524

ATP binding

Molecular Function

Definition

Binding to ATP, adenosine 5'-triphosphate, a universally important coenzyme and enzyme regulator.

GO:0030424

Axon

Cellular Component

Definition

The long process of a neuron that conducts nerve impulses, usually away from the cell body to the terminals and varicosities, which are sites of storage and release of neurotransmitter.

GO:0005515

protein binding

Molecular Function

Definition

Binding to a protein.

**01**

GO:0007268

chemical synaptic transmission

Biological Process

Definition

The vesicular release of classical neurotransmitter molecules from a presynapse, across a chemical synapse, the subsequent activation of neurotransmitter receptors at the postsynapse of a target cell (neuron, muscle, or secretory cell) and the effects of this activation on the postsynaptic membrane potential and ionic composition of the postsynaptic cytosol. This process encompasses both spontaneous and evoked release of neurotransmitter and all parts of synaptic vesicle exocytosis. Evoked transmission starts with the arrival of an action potential at the presynapse.

Keywords - Cell membrane (KW-1003)

Protein found in or associated with the cytoplasmic membrane, a selectively permeable membrane which separates the cytoplasm from its surroundings. Known as the cell inner membrane in prokaryotes with 2 membranes.

GO:0005886

plasma membrane

Cellular Component

Definition

The membrane surrounding a cell that separates the cell from its external environment. It consists of a phospholipid bilayer and associated proteins.

**02**

SH3 (src Homology-3) domains are small protein modules containing approximately 50 amino acid residues [1, 2]. They are found in a great variety of intracellular or membrane-associated proteins [3, 4, 5] for example, in a variety of proteins with enzymatic activity, in adaptor proteins, such as fodrin and yeast actin binding protein ABP-1.

-The SH3 domain has a characteristic fold which consists of five or six β-strands arranged as two tightly packed anti-parallel β-sheets. The linker regions may contain short helices. The surface of the SH3-domain bears a flat, hydrophobic ligand-binding pocket which consists of three shallow grooves defined by conservative aromatic residues in which the ligand adopts an extended left-handed helical arrangement. The ligand binds with low affinity but this may be enhanced by multiple interactions. The region bound by the SH3 domain is in all cases proline-rich and contains PXXP as a core-conserved binding motif. The function of the SH3 domain is not well understood but they may mediate many diverse processes such as increasing local concentration of proteins, altering their subcellular location and mediating the assembly of large multiprotein complexes [6

**03**

CAMP signaling pathways,

cAMP signaling pathways (cAMP signal pathway) in cAMP signaling pathways, G alpha is the primary effect of enzyme adenylate cyclase (adenylyl cyclase (AC), by the activity of adenylate cyclase change adjust the level of target intracellular second messenger cAMP, And then affect the downstream events of the signaling pathway.

**04**

None

**05**

None

**06**

GO:0005524

ATP binding

Molecular Function

Definition

Binding to ATP, adenosine 5'-triphosphate, a universally important coenzyme and enzyme regulator.

GO:0043087

regulation of GTPase activity

Biological Process

Definition

Any process that modulates the rate of GTP hydrolysis by a GTPase.

GO:0004896

cytokine receptor activity

Molecular Function

Definition

Combining with a cytokine and transmitting the signal from one side of the membrane to the other to initiate a change in cell activity.

GO:0019888

protein phosphatase regulator activity

Molecular Function

Definition

Binds to and modulates the activity of a protein phosphatase.

GO:0071526

semaphorin-plexin signaling pathway

Biological Process

Definition

The series of molecular signals generated as a consequence of a semaphorin receptor (composed of a plexin and a neurophilin) binding to a semaphorin ligand. PMID:15239959

GO:0030424

Axon

Cellular Component

Definition

The long process of a neuron that conducts nerve impulses, usually away from the cell body to the terminals and varicosities, which are sites of storage and release of neurotransmitter.

GO:0016020

Membrane

Cellular Component

Definition

A lipid bilayer along with all the proteins and protein complexes embedded in it an attached to it.

Human papillomavirus infection pathway

（ATP6AP1,BCAP31,CREBBP,WNT5B,CREB5,DLG3,EGFR,IKBKG,ITGA4,ITGA5,ITGA8,MTOR,PARD3,PDGFRB,PTGS2,PPP2R3B,PPP2R5D,PPP2R5C,PPP2R2B,THBS2,)

GO:0043552

positive regulation of phosphatidylinositol 3-kinase activity

Biological Process

Definition

Any process that activates or increases the frequency, rate or extent of phosphatidylinositol 3-kinase activity.

GO:0032991

protein-containing complex

Cellular Component

Definition

A stable assembly of two or more macromolecules, i.e. proteins, nucleic acids, carbohydrates or lipids, in which at least one component is a protein and the constituent parts function together.

GO:0030426

growth cone

Cellular Component

Definition

The migrating motile tip of a growing neuron projection, where actin accumulates, and the actin cytoskeleton is the most dynamic. PMID:10082468

GO:0050790

regulation of catalytic activity

Biological Process

Definition

Any process that modulates the activity of an enzyme.

GO:0045944

positive regulation of transcription by RNA polymerase II

Biological Process

Definition

Any process that activates or increases the frequency, rate or extent of transcription from an RNA polymerase II promoter.

GO:0000122

negative regulation of transcription by RNA polymerase II

Biological Process

Definition

Any process that stops, prevents, or reduces the frequency, rate or extent of transcription mediated by RNA polymerase II

GO:0004672

protein kinase activity

Molecular Function

Definition

Catalysis of the phosphorylation of an amino acid residue in a protein, usually according to the reaction: a protein + ATP = a phosphoprotein + ADP.

GO:0008285

negative regulation of cell population proliferation

Biological Process

Definition

Any process that stops, prevents or reduces the rate or extent of cell proliferation.

Pathway :Proteoglycans in cancer

（ARAF,SRC,WNT5B,EGFR,ERBB4,ESR1,FLNA,IGF1R,ITGA5,MTOR,MSN,PTCH1,SDC1,TGFB1,VAV2）

GO:0050808

synapse organization

Biological Process

Definition

A process that is carried out at the cellular level which results in the assembly, arrangement of constituent parts, or disassembly of a synapse, the junction between a neuron and a target (neuron, muscle, or secretory cell).

GO:0004714

transmembrane receptor protein tyrosine kinase activity

Molecular Function

Definition

Combining with a signal and transmitting the signal from one side of the membrane to the other to initiate a change in cell activity by catalysis of the reaction: ATP + a protein-L-tyrosine = ADP + a protein-L-tyrosine phosphate.

GO:0004713

protein tyrosine kinase activity

Molecular Function

Definition

Catalysis of the reaction: ATP + a protein tyrosine = ADP + protein tyrosine phosphate.

GO:0019901

protein kinase binding

Molecular Function

Definition

Binding to a protein kinase, any enzyme that catalyzes the transfer of a phosphate group, usually from ATP, to a protein substrate.

MAPK signaling pathway

(ARAF,RELB,RASGRF1,CACNB4,CACNA1F,CDC25B,DUSP9,EGFR,ERBB4,FGF3,FLNA,FLT3,IKBKG,IGF1R,IRAK1,MAPT,MAP2K6,MAP2K7,NF1,PDGFRB,TGFB1）

GO:0007165

signal transduction

Biological Process

Definition

The cellular process in which a signal is conveyed to trigger a change in the activity or state of a cell. Signal transduction begins with reception of a signal (e.g. a ligand binding to a receptor or receptor activation by a stimulus such as light), or for signal transduction in the absence of ligand, signal-withdrawal or the activity of a constitutively active receptor. Signal transduction ends with regulation of a downstream cellular process, e.g. regulation of transcription or regulation of a metabolic process. Signal transduction covers signaling from receptors located on the surface of the cell and signaling via molecules located within the cell. For signaling between cells, signal transduction is restricted to events at and within the receiving cell.

PI3K-Akt signaling pathway

（GNGT1,CREB5,EGFR,ERBB4,FGF3,FLT3,IKBKG,IGF1R,ITGA4,ITGA5,ITGA8,IL2RG,IL3RA,IL7R,MTOR,PDGFRB,PPP2R3B,PPP2R5D,PPP2R2B,RXRA,STK11,SYK,THBS2）

GO:0019899

enzyme binding

Molecular Function

Definition

Binding to an enzyme, a protein with catalytic activity

GO:0005634

Nucleus

Cellular Component

Definition

A membrane-bounded organelle of eukaryotic cells in which chromosomes are housed and replicated. In most cells, the nucleus contains all of the cell's chromosomes except the organellar chromosomes, and is the site of RNA synthesis and processing. In some species, or in specialized cell types, RNA metabolism or DNA replication may be absent.

**07**

PDZ domain

PDZ domains (also known as Discs-large homologous regions (DHR) or GLGF)) are found in diverse signalling proteins in bacteria, yeasts, plants, insects and vertebrates [1, 2]. PDZ domains can occur in one or multiple copies and are nearly always found in cytoplasmic proteins. They bind either the carboxyl-terminal sequences of proteins or internal peptide sequences [2]. In most cases, interaction between a PDZ domain and its target is constitutive, with a binding affinity of 1 to 10 microns. However, agonist-dependent activation of cell surface receptors is sometimes required to promote interaction with a PDZ protein. PDZ domain proteins are frequently associated with the plasma membrane, a compartment where high concentrations of phosphatidylinositol 4,5-bisphosphate (PIP2) are found. Direct interaction between PIP2 and a subset of class II PDZ domains (syntenin, CASK, Tiam-1) has been demonstrated.

PDZ domains consist of 80 to 90 amino acids comprising six β-strands (beta-A to beta-F) and two α-helices, A and B, compactly arranged in a globular structure. Peptide binding of the ligand takes place in an elongated surface groove as an anti-parallel β-strand interacts with the beta-B strand and the B helix. The structure of PDZ domains allows binding to a free carboxylate group at the end of a peptide through a carboxylate-binding loop between the beta-A and beta-B strands [3].

Hippo signaling pathway(DLG2,PARD6B,PARD6G)

Tight junction PATHWAY(DLG2,PARD6B,PARD6G,)

GO:0060341

regulation of cellular localization

Biological Process

Definition (GO:0060341 GONUTS page)

Any process that modulates the frequency, rate or extent of a process in which a cell, a substance, or a cellular entity is transported to, or maintained in a specific location within or in the membrane of a cell.

Human papillomavirus infection(DLG2,PARD6B,PARD6G)

PB1 domain

Description: Phox and Bem1p domain, present in many eukaryotic cytoplasmic signalling proteins. The domain adopts a beta-grasp fold, similar to that found in ubiquitin and Ras-binding domains. A motif, variously termed OPR, PC and AID, represents the most conserved region of the majority of PB1 domains, and is necessary for PB1 domain function. This function is the formation of PB1 domain heterodimers, although not all PB1 domain pairs associate.

**08**

Keywords - DNA repair (KW-0234)

Protein involved in the repair of DNA, the various biochemical processes by which damaged DNA can be restored. DNA repair embraces, for instance, not only the direct reversal of some types of damage (such as the enzymatic photoreactivation of thymine dimers), but also multiple distinct mechanisms for excising damaged base; termed nucleotide excision repair (NER), base excision repair (BER) and mismatch repair (MMR); or mechanisms for repairing double-strand breaks.

Keywords - DNA damage (KW-0227)

Protein induced by DNA damage or protein involved in the response to DNA damage. Drug- or radiation-induced injuries in DNA introduce deviations from its normal double-helical conformation. These changes include structural distortions which interfere with replication and transcription, as well as point mutations which disrupt base pairs and exert damaging effects on future generations through changes in DNA sequence. Response to DNA damage results in either repair or tolerance.

GO:0005634

Nucleus

Cellular Component

Definition

A membrane-bounded organelle of eukaryotic cells in which chromosomes are housed and replicated. In most cells, the nucleus contains all of the cell's chromosomes except the organellar chromosomes, and is the site of RNA synthesis and processing. In some species, or in specialized cell types, RNA metabolism or DNA replication may be absent.

**09**

GO:0034704

calcium channel complex

Cellular Component

Definition

An ion channel complex through which calcium ions pass.

GO:0005891

voltage-gated calcium channel complex

Cellular Component

Definition

A protein complex that forms a transmembrane channel through which calcium ions may pass in response to changes in membrane potential.

GO:0016529

sarcoplasmic reticulum

Cellular Component

Definition

A fine reticular network of membrane-limited elements that pervades the sarcoplasm of a muscle cell; continuous over large portions of the cell and with the nuclear envelope; that part of the endoplasmic reticulum specialized for calcium release, uptake and storage.

Keywords - Calcium transport (KW-0109)

Protein involved in the transport of calcium ions. Calcium is essential for a variety of bodily functions, such as neurotransmission, muscle contraction and proper heart function.

GO:0006816

calcium ion transport

Biological Process

Definition

The directed movement of calcium (Ca) ions into, out of or within a cell, or between cells, by means of some agent such as a transporter or pore.

**10**

regulation of DNA-templated transcription

Biological Process

Definition

Any process that modulates the frequency, rate or extent of cellular DNA-templated transcription

GO:0006357

regulation of transcription by RNA polymerase II

Biological Process

Definition

Any process that modulates the frequency, rate or extent of transcription mediated by RNA polymerase II.

**11**

GO:0030424

Axon

Cellular Component

Definition

The long process of a neuron that conducts nerve impulses, usually away from the cell body to the terminals and varicosities, which are sites of storage and release of neurotransmitter.

GO:0009986

cell surface

Cellular Component

Definition

The external part of the cell wall and/or plasma membrane.

Keywords - Autism (KW-1269)

Protein which, if defective, is involved in autism, a pervasive developmental disorder. It is a complex, multifactorial disease characterized by impairments in reciprocal social interaction and communication, restricted and stereotyped patterns of interests and activities, and the presence of developmental abnormalities by 3 years of age. Most individuals with autism manifest moderate mental retardation.

Keywords - Autism spectrum disorder (KW-1268)

Protein which, if defective, is involved in autism spectrum disorder, a clinically heterogeneous group of disorders that share common features of impaired social relationships, impaired language and communication, repetitive behaviors, and a restricted range of interests. The spectrum includes diverse phenotypic manifestations, such as classic autism, Asperger syndrome, childhood disintegrative disorder, Rett syndrome, and pervasive developmental disorder not otherwise specified.

Keywords - Cell junction (KW-0965)

Protein found in or associated with a cell junction, a cell-cell or cell-extracellular matrix contact within a tissue of a multicellular organism, especially abundant in epithelia. In vertebrates, there are three major types of cell junctions: anchoring junctions (e.g. adherens junctions), communicating junctions (e.g. gap junctions) and occluding junctions (e.g. tight junctions).

GO:0097105

presynaptic membrane assembly

Biological Process

Definition

The aggregation, arrangement and bonding together of a set of components to form a presynaptic membrane, including any proteins associated with the membrane, but excluding other cellular components. A presynaptic membrane is a specialized area of membrane of the axon terminal that faces the plasma membrane of the neuron or muscle fiber with which the axon terminal establishes a synaptic junction. PMID:15797875 PMID:18550748

**12**

Keywords - Nucleus (KW-0539)

Protein located in the nucleus of a cell.

**13**

GO:0034198

cellular response to amino acid starvation

Biological Process

Definition

Any process that results in a change in state or activity of a cell (in terms of movement, secretion, enzyme production, gene expression, etc.) as a result of deprivation of amino acids

HIF-1 signaling pathway(TEK,HIF1A)

HLH (helix loop helix domain)

A number of eukaryotic proteins, which probably are sequence specific DNA- binding proteins that act as transcription factors, share a conserved domain of 40 to 50 amino acid residues. It has been proposed [ (PUBMED:2493990) ] that this domain is formed of two amphipathic helices joined by a variable length linker region that could form a loop. This 'helix-loop-helix' (HLH) domain mediates protein dimerization and has been found in the proteins listed below [ (PUBMED:1521738) ]. Most of these proteins have an extra basic region of about 15 amino acid residues that is adjacent to the HLH domain and specifically binds to DNA. They are refered as basic helix-loop-helix proteins (bHLH), and are classified in two groups: class A (ubiquitous) and class B (tissue-specific). Members of the bHLH family bind variations on the core sequence 'CANNTG', also refered to as the E-box motif. The homo- or heterodimerization mediated by the HLH domain is independent of, but necessary for DNA binding, as two basic regions are required for DNA binding activity. The HLH proteins lacking the basic domain (Emc, Id) function as negative regulators, since they form heterodimers, but fail to bind DNA. The hairy-related proteins (hairy, E(spl), deadpan) also repress transcription although they can bind DNA. The proteins of this subfamily act together with co-repressor proteins, like groucho, through their C-terminal motif WRPW.

Pathway:Autophagy – animal (RRAGB,HIF1A)

Pathway:mTOR signaling pathway(RRAGB,RNF152)

FN3(Fibronectin type 3 domain)

One of three types of internal repeat within the plasma protein, fibronectin. The tenth fibronectin type III repeat contains a RGD cell recognition sequence in a flexible loop between 2 strands. Type III modules are present in both extracellular and intracellular proteins.

**14**

GO:0005524

ATP binding

Molecular Function

Definition

Binding to ATP, adenosine 5'-triphosphate, a universally important coenzyme and enzyme regulator.

Keywords - ATP-binding (KW-0067)

Definition

Protein which binds adenosine 5'-triphosphate (ATP), a ribonucleotide adenosine (a purine base adenine linked to the sugar D-ribofuranose) that carries three phosphate groups esterified to the sugar moiety. It is the cell's source for energy and phosphate.

GO:0000794

condensed nuclear chromosome

Cellular Component

Definition

A highly compacted molecule of DNA and associated proteins resulting in a cytologically distinct nuclear chromosome.

Keywords - Nucleotide-binding (KW-0547)

Definition

Protein which binds a nucleotide, a phosphate ester of a nucleoside consisting of a purine or pyrimidine base linked to ribose or deoxyribose phosphates.

P-loop containing nucleoside triphosphate hydrolase

The P-loop NTPase fold is the most prevalent domain of the several distinct nucleotide-binding protein folds.

The most common reaction catalysed by enzymes of the P-loop NTPase fold is the hydrolysis of the beta-gamma phosphate bond of a bound nucleoside triphosphate (NTP). The energy from NTP hydrolysis is typically utilised to induce conformational changes in other molecules, which constitutes the basis of the biological functions of most P-loop NTPases. P-loop NTPases show substantial substrate preference for either ATP or GTP

P-loop NTPases are characterised by two conserved sequence signatures, the Walker A motif (the P-loop proper) and Walker B motifs which bind, respectively, the beta and gamma phosphate moieties of the bound NTP, and a Mg2+ cation.

P-loop ATPase domains belong to one of the two major divisions. The kinase-GTPase (KG) division includes the kinases and GTPases, and the ASCE division, characterised by an additional strand in the core sheet, which is located between the P-loop strand and the Walker B strand. Most members of the ASCE division utilise ATP and members of this group include AAA+, ABC, PilT, HerA-FtsK, superfamily 1/2 (SF1/2) helicases, and the RecA/ATP-synthase superfamilies of ATPases, etc

GO:0016887

ATP hydrolysis activity

Molecular Function

Definition

Catalysis of the reaction: ATP + H2O = ADP + H+ phosphate. ATP hydrolysis is used in some reactions as an energy source, for example to catalyze a reaction or drive transport against a concentration gradient.

**15**

Pathway:Inositol phosphate metabolism

(INPP5F,MTMR1,PIK3C2G)

Pathway:Phosphatidylinositol signaling system

(INPP5F,MTMR1,PIK3C2G)

GO:0046856 JSON

phosphatidylinositol dephosphorylation

Biological Process

Definition

The process of removing one or more phosphate groups from a phosphatidylinositol.

GO:0006661 JSON

phosphatidylinositol biosynthetic process

Biological Process

Definition

The chemical reactions and pathways resulting in the formation of phosphatidylinositol, any glycophospholipid in which the sn-glycerol 3-phosphate residue is esterified to the 1-hydroxyl group of 1D-myo-inositol.

GO:0048015 JSON

phosphatidylinositol-mediated signaling

Biological Process

Definition

The series of molecular signals in which a cell uses a phosphatidylinositol-mediated signaling to convert a signal into a response. Phosphatidylinositols include phosphatidylinositol (PtdIns) and its phosphorylated derivatives.

Pathway:Metabolic pathways

(INPP5F,MTMR1,PIK3C2G)

Keywords - Lipid metabolism (KW-0443)

Definition

Protein involved in the biochemical reactions of lipids. Lipids are a diverse class of compounds which are insoluble in water but soluble in organic solvents. They include fats, oils, triacylglycerols, fatty acids, glycolipids, phospholipids and steroids.

**16**

Pathway:Apoptotic DNA fragmentation and tissue homeostasis

(TOP2B,CASP3)

**17**

GO:0048010

vascular endothelial growth factor receptor signaling pathway

Biological Process

Definition

The series of molecular signals initiated by a ligand binding to a vascular endothelial growth factor receptor (VEGFR) on the surface of the target cell, and ending with the regulation of a downstream cellular process, e.g. transcription.

Serine-threonine/tyrosine-protein kinase, catalytic domain

Description

Protein phosphorylation, which plays a key role in most cellular activities, is a reversible process mediated by protein kinases and phosphoprotein phosphatases. Protein kinases catalyse the transfer of the gamma phosphate from nucleotide triphosphates (often ATP) to one or more amino acid residues in a protein substrate side chain, resulting in a conformational change affecting protein function. Phosphoprotein phosphatases catalyse the reverse process. Protein kinases fall into three broad classes, characterised with respect to substrate specificity

Pathway:Rap1 signaling pathway

**18**

GO:0003677

DNA binding

Molecular Function

Definition

Any molecular function by which a gene product interacts selectively and non-covalently with DNA (deoxyribonucleic acid).

Keywords - Ubl conjugation (KW-0832)

Definition

Protein which is posttranslationally modified by the attachment of at least one ubiquitin-like modifier protein, such as ubiquitin, SUMO, APG12, URM1 or RUB1. Ubiquitin, for example, is linked through a isopeptide bond between its C-terminus and the epsilon group of a lysine residue present on either another ubiquitin-like modifier protein or a target protein.

GO:0005634

Nucleus

Cellular Component

Definition (GO:0005634 GONUTS page)

A membrane-bounded organelle of eukaryotic cells in which chromosomes are housed and replicated. In most cells, the nucleus contains all of the cell's chromosomes except the organellar chromosomes, and is the site of RNA synthesis and processing. In some species, or in specialized cell types, RNA metabolism or DNA replication may be absent.
